# Deep Learning Applied to Vegetation Identification and Removal Using Multidimensional Aerial Data

**DOI:** 10.3390/s20216187

**Published:** 2020-10-30

**Authors:** Milena F. Pinto, Aurelio G. Melo, Leonardo M. Honório, André L. M. Marcato, André G. S. Conceição, Amanda O. Timotheo

**Affiliations:** 1 Department of Electronics Engineering, Federal Center for Technological Education of Rio de Janeiro, Rio de Janeiro 20260-100, Brazil; milena.pinto@cefet-rj.br; 2 Department of Electrical Engineering, Federal University of Juiz de Fora, Juiz de Fora 36073-120, Brazil; aurelio.melo@engenharia.ufjf.br (A.G.M.); andre.marcato@engenharia.ufjf.br (A.L.M.M.); amanda.timotheo@engenharia.ufjf.br (A.O.T.); 3 Department of Electrical Engineering, Federal University of Bahia, Salvador 40210-630, Brazil; andre.gustavo@ufba.br

**Keywords:** vegetation identification/recognition, 3D point cloud, deep learning, Unmanned Aerial Vehicles, structural analyzes

## Abstract

When performing structural inspection, the generation of three-dimensional (3D) point clouds is a common resource. Those are usually generated from photogrammetry or through laser scan techniques. However, a significant drawback for complete inspection is the presence of covering vegetation, hiding possible structural problems, and making difficult the acquisition of proper object surfaces in order to provide a reliable diagnostic. Therefore, this research’s main contribution is developing an effective vegetation removal methodology through the use of a deep learning structure that is capable of identifying and extracting covering vegetation in 3D point clouds. The proposed approach uses pre and post-processing filtering stages that take advantage of colored point clouds, if they are available, or operate independently. The results showed high classification accuracy and good effectiveness when compared with similar methods in the literature. After this step, if color is available, then a color filter is applied, enhancing the results obtained. Besides, the results are analyzed in light of real Structure From Motion (SFM) reconstruction data, which further validates the proposed method. This research also presented a colored point cloud library of bushes built for the work used by other studies in the field.

## 1. Introduction

Many techniques can be applied in order to monitor different aspects of the terrain when inspecting large structures. For instance, photogrammetry is a widely used method, due to its flexibility, cost-effectiveness, and accuracy [1]. This technique can be applied in many different cases, such as presented in Barazzetti et al. [2], and it can be easily combined with other technologies to improve the results. For instance, 3D reconstruction by photogrammetry combined with Unmanned Aerial Vehicles (UAVs) results in a very dense point cloud with very low costs and surveying time [3,4]. Works of Khaloo et al. [5] and Pinto et al. [6] showed a potential use of UAV along with photogrammetry to perform large structures’ inspection. This kind of process generates point clouds with acurracy. Another similar approach is presented by Buffi et al. [7], where the images are also georeferenced by applying Ground Control Points (GCP) to the inspected structure, further increasing inspection accuracy [8].

Despite the promising results, many issues may arise during the post-processing stage of image analyses. A significant problem for determining structural problems in massive constructions, like slopes and dams, is the presence of areas that are covered by shrubs and vegetation. Note that vegetation can cover large portions of the inspected surface, which does not allow for an adequate acquisition of the object surface. This is a significant disadvantage in surface modeling applications for geomorphological analysis [9].

Note that the presence of vegetation is even more critical in applications that involve monitoring tasks, such as in Abellan et al. [10], where vegetation interferes with the surfaces’ shapes. Thus, the problem of vegetation removal has to be provided as a post-processing stage to effectively accomplish inspections of structures. A few works have already addressed this issue by applying machine learning and other techniques, such as in [11,12,13,14,15]. However, there is the necessity of more general methods that can be easily applied in different conditions after a single training session.

There are some techniques for classifying and recognizing vegetation features [16,17]. The authors in Natrajan et al. [18] proposed the classification of hyperspectral images of crops while using Convolutional Neural Network (CNN). They identified regions that contain grapes samples using CNN, and transfer learning in order to overcome data that is not labeled. A drawback of this approach is that the hyperspectral camera also identifies moss as vegetation, significantly reducing the point-cloud density. Experimental results from this method proved that patching images are capable of training better CNN. However, computational costs can increase considerably if a larger patch size is used for CNN. As stated by [19], an advantage of CNN application is the use of local connections in order to extract spatial information and shared weights to reduce the number of parameters.

Several pieces of research have developed methods considering vegetation removal. For example, Zhao et al. [20] proposed a new algorithm based on Reference Spectral Background Removal (RSBR), capable of extracting the high-density plants’ area by removing the image background. The work of Sithole et al. [21] presented the development of an algorithm for extracting bare earth from point clouds. The data set was obtained by a laser scanner and tested by filter algorithms. Reference data tools were used in order to remove points of the vegetation. Their results were satisfactory when performed in soft rural areas. However, their technique produced errors in rugged terrains, such as mining slopes.

A practical application of vegetation removal in natural environments is found in Brodu and Lague [22], where the vegetation is recognized with high accuracy through a classification method for 3D point clouds that are explicitly designed for complex natural environments. Their algorithm is called CANUPO suit. The algorithm CANUPO uses a Support Vector Machine (SVM) in order to perform the classification. In some aspects, such as computational cost in training and performance, the SVM application is a good strategy. However, it has some shortfalls, such as scalability to recognize multiples types of classes and low performance without user intervention.

The PointNet proposed by Qi et al. [23] is another similar work. It is important to mention that this strategy presents a similar structure to the one proposed by this work. However, PointNet cannot deal with color data. Color information may improve performance for applications, such as Structure From Motion (SFM) reconstructions, where color is usually available at the reconstructions. SFM is a technique used to estimate three-dimensional (3D) structures from sequences of two-dimensional images. Note that this technique only requires a camera. Thus, the costs are significantly lower than the approaches that use lasers along with photogrammetry [24,25]. However, SFM reconstructions present greater errors in measurements. For more comparison information, the authors refer to [26].

Therefore, the main focus of this research is to develop a methodology that is capable of removing the covering vegetation and also generates the underlying structure. The vegetation removal must be precise and minimal to use only well classified remaining points to reconstruct the original surface and then, to monitor the aspects of the structure. This work develops a machine learning methodology to directly classify vegetation using 3D information in the point cloud data. The processing steps are demonstrated along with training results and the basic built library of bushes provided by the authors. This work also provides results for real-world reconstructions of dams and slopes. The contributions can be summarized, as follows:

- A robust method for vegetation identification and minimal removal from 3D point cloud data.
- A real application of natural scene removal for slopes and dams inspection.

The remainder of this work is organized, as follows. Section 2 details the proposed methodology and its mathematical foundations. Section 3 shows the proposed experiments with a proper discussion of the results. Section 4 illustrates the concluding remarks and future work.

## 2. Methodology for 3D Data Classification of Complex Natural Scenes

There are many approaches in the literature for image classification and object position estimation. The most accurate methods for classification apply state-of-the-art deep learning structures, and most of those methods can also be applied to 3D point clouds [27]. Sometimes, classification is not enough to determine object position in a given image, requiring, in this sense, additional algorithms. For instance, the Bag of Words (BOW) method [28] presents a stage of sliding window processing. This process takes consecutive segments of the image and applies the classification method in each window, increasing the computational cost and limiting the efficiency of the position estimation in the windowing step. Despite those disadvantages, there is an accuracy increase that is related to the size standardization promoted by the windowing stage [29].

### 2.1. Framework Overview

**Figure 1:**
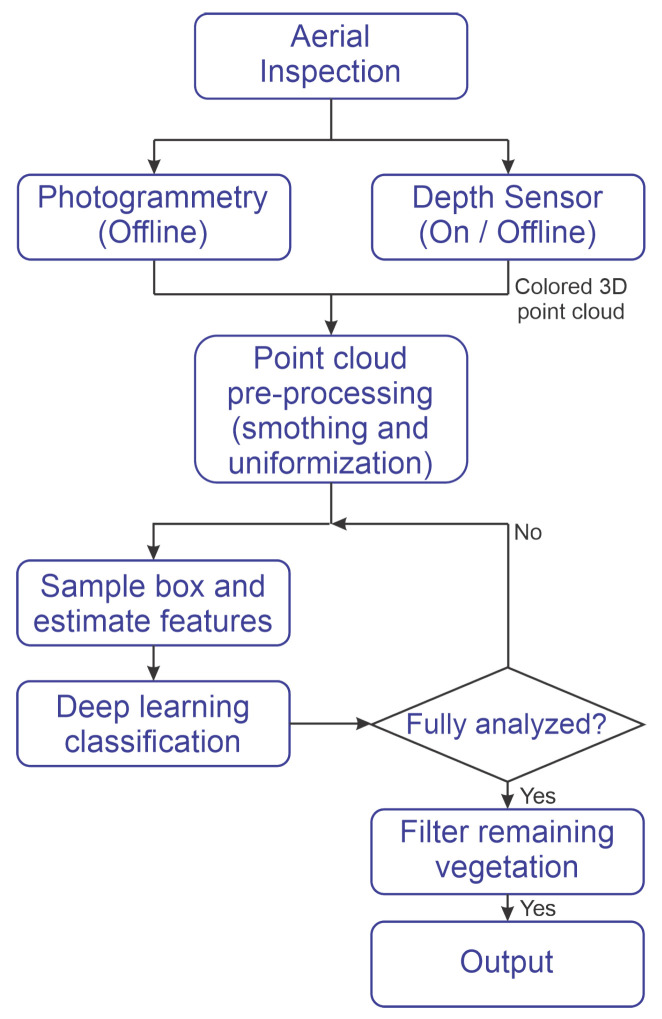
Methodology Diagram.

Figure 1 represents the proposed process. The input of this process is a 3D point cloud. Note that this point cloud can be generated, whether by SFM or by the deployment of sensors, such as stereo and depth cameras. This resultant point cloud is pre-processed in a filter stage to smooth and uniform the data, allowing for an accurate classification in the next process. The output data from the pre-processing stage is segmented into boxes, and a feature extraction method is applied at each box. Subsequently, a deep learning method classifies the contents of each box. In case that vegetation is perceived inside a box, its content is removed from the main file. A final filtering stage is used in order to remove the remaining vegetation parts.

In a regular image, the data are uniformly distributed. This means that the amount of pixels in a given stretch of the image is constant. However, this is not a valid proposition for a point cloud. For instance, photogrammetric reconstruction tends to generate large amounts of data at the center of the reconstruction, and sparser at the edges where the overlapping may be less concise, a more detailed analisys can be seen in [30]. This is a problem for the classification process. Therefore, the first step is to smooth the point cloud by applying a low distance filter. The filter works by fitting hyperplanes of defined size throughout the point cloud, as shown in Equation (1).

(1) $$\beta_{0}+\beta_{1}\cdot X_{1}+\ldots +\beta_{P}\cdot X_{P}$$

### 2.2. SFM Point Clouds Preprocessing

**Figure 2:**
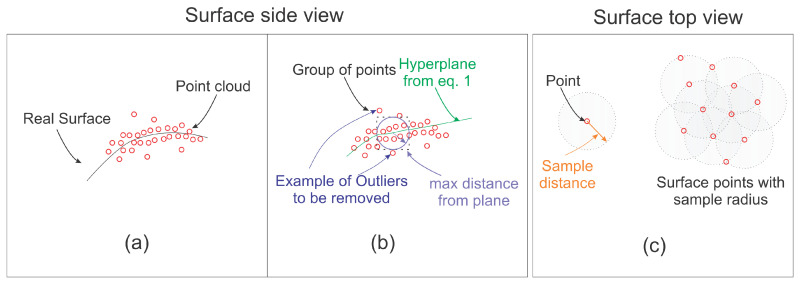
Ilustration of the filtering process (**a**) real surface and reconstruction (**b**) local hyperplane fitting and (**c**) uniform sampling.

Subsequently, the filter removes points if their distances to the plane are more significant than a given threshold ($d_{\varepsilon }$). Afterwards, the parameter β is estimated while using the threshold $d_{\varepsilon }$. Another processing stage is applied after smoothing the point data and making its distribution uniform. The dense cloud needs to be sub-sampled to a sparse cloud. Note that a high amount of points would be good for a classification once it would have more available details. However, a large number of points require higher processing power at the training stage. This could also result in a huge memory requirement, making the classifier algorithm more challenging to be trained without improving the results significantly. Figure 2 illustrate the process described. The input cloud is shown in Figure 2a with the real surface represented. In Figure 2, a group of points is selected, as shown by the bound box. A hyperplane represented in green is estimated and points that are too far from this hyperplane inside the local region are removed. Such points are exemplified in Figure 2b by the blue arrow. After this step, Figure 2c illustrates the surface as seen from above. In this part of the figure, grey circles indicate the sampling radius. Duplicated points inside this radius are removed, making the sampling more uniform.

The sparse cloud that was obtained from the reconstruction contains a 3D position and RGB color. The RGB information can be compressed into a single luminosity channel for memory optimization, which is represented by (Lum). The selected transformation was the CIR 1991 colorimetric models proposed by [31] and presented in Equation (2). This equation inputs the color information for each pixel and converts it into single monochromatic luminosity information. Note that the green channel (G) is privileged over red (R) and blue (B), once it is the most common color in bushes.

(2) Lum=R·0.2126+G·0.7152+B·0.0722

Subsequently, this luminosity channel is compressed into a histogram that is used as a parameter in the classification process. This process is performed by estimating the histogram for luminosity values into eight classes. Each class will represent the frequency for 32 luminosity values, as the luminosity channel can assume values between 0 and 255.

The point positions are also transformed into a feature space while using Fast Point Feature Histogram (FPFH) [32] presented in the Point Cloud Library (PCL). The process is performed by estimating geometric relationships for the point-sets and then grouping them into feature histograms, reducing the amount of information that machine learning has to deal with.

### 2.3. Vegetation Identification

#### 2.3.1. Feature Extraction and Classification

Feature descriptors, such as this one, are, in general, very robust to transformation changes. In this work, the point cloud’s orientation and its position were not particularly selected to comply with any axis relationship. Works, such [33], have shown that this feature descriptor is very suitable to use in conditions with changes in point cloud orientation, still presenting good performance.

**Figure 3:**
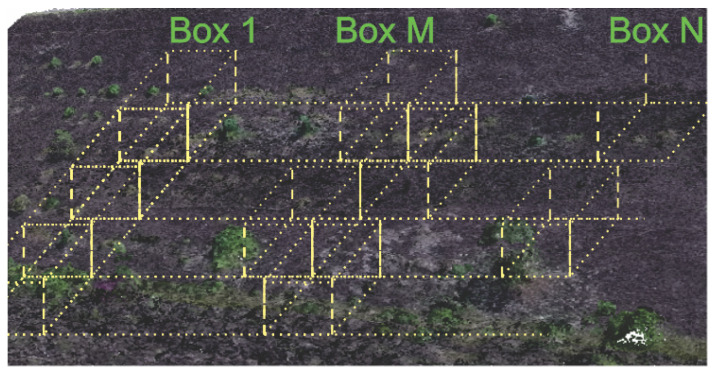
Boxing Process.

As described, the classification occurs by selecting a box of the data and applying the deep learning trained algorithm. After, the box is moved to the next location and the classification process is applied. This process should be repeated until the point cloud is thoroughly analyzed. Figure 3 shows a representation of the boxing process.

After the classification in each box, there is another filtering step. This step intends to remove vegetation points that were leftover from the classification process. In this process, the average RGB color from the classified vegetation is estimated. After every point that has a color similar enough within a given threshold and bounded distance, the algorithm removes the vegetation points. Equations (3) and (4) show the point removal process. Basically, Equation (3) estimates a distance for a given point in relation to its neighbor. Then, Equation (4) determines if the point will be eliminated based on its color and distance. The variable $p_{i}a$ is considered as a position of the *i*th point in the “*a*” axis of the point cloud *P*. The variable $\tau_{d}$ is the maximum distance from the vegetation neighbor points.

(3) $$d\left(p_{1},p_{2}\right)=\sqrt{\sum {\left(p_{1a}-p_{2a}\right)}^{2}}$$

(4) $$min\;d\left(p_{1},p_{i}\right)\;\forall p\;\left\{p_{i}\in \;P\;and\;p_{1}\neq p_{i}\right\}<\tau_{d}$$

For the points that fit in Equations (3) and (4), it is possible to verify color similarity, as previously defined. The variable $c_{ia}$ is the i-th point color, where ‘*a*’ is the value of the red, green, or blue channel in RGB. Equation (5) presents this color distance estimation. Note that the points that are at a lower distance than the color threshold $\tau_{c}$ are removed.

(5) $$d\left(c_{i},c_{avg}\right)=\sqrt{\sum_{\forall a}\left(c_{ia}-c_{\left(avga\right)}\right)}$$

Algorithm 1 summarizes all of the processes described in this section. This algorithm details the input and outputs of the method as well as the steps that are taken. Note that a few more steps, such as normal estimation, are required. All of those steps were implemented in PCL using C++.
**Algorithm 1** Vegetation Extraction Algorithm**Input:** 3D point cloud to be processed;
  1: Split point cloud into boxes;  2: **for** each box  3:  Estimate threshold $\tau_{d}$ and apply point filter;  4:  Compress $c_{i}$ color channels in Lum channel;  5:  Center point cloud $p_{i}$;  6:  Resample point cloud $p_{i}$ to normalize point density;  7:  Estimate normals $n_{pi}$ for point cloud $p_{i}$;  8:  Estimate FPFH histogram and Lum color histogram;  9:  Select randomly 200 histograms;  10:   Save histogram file;  11:   Perform Classification;  12:   Remove classified vegetation for a separated file;  13:**end**  14:Estimate mean color of the removed vegetation;  15:Filter points with similar colors in the final point cloud;
**Output:** Point cloud with vegetation removed;**Output:** Vegetation files;

#### 2.3.2. Neural Network Model Training

**Figure 4:**
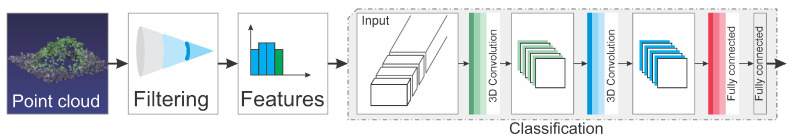
Proposed Neural Network Architecture for Classification.

The design of the neural network was selected based on other image processing networks. After some experimentation, this design was refined to produce proper performance. The neural network structure is formed by three main components, where two sets of layers are at the core interconnected by max-pooling layers and fully connected layers at the end. Figure 4 illustrates this structure. Because classification is the main objective, the network output is a score that represents the class certainty.

After the network design, one of the most critical steps in machine learning algorithms is the training stage. Thus, a good database containing trained cases is a requirement to produce accurate results. Some algorithms, such as SVM, need fewer examples. However, other ones, like neural networks, may require more examples to produce accurate results. Therefore, a database containing 57 bushes examples was built in order to fulfill the training process. The other three classes from ModelNet40 [34] and SIGGRAPH [35] databases (e.g., car, people, and road) containing 200 examples of each object was used to provide a control classification of the model.

Defining the database is not the only important task. Several issues may arise when dealing with 3D information in the point cloud. For instance, the Point of View (PoV) means that the 3D information may change significantly according to the camera orientation, which becomes extremely relevant in real-time applications. However, for 3D photogrammetric tasks, this issue is less dramatic once the 3D reconstruction should always contain information about the 360-degree view of the object. Therefore, the database was augmented by applying rotation, scaling, and crop to training samples in order to improve classification results, which also improves the outcomes once a bush can split among two boxes.

The database was used to train the neural network. Note that real reconstructions from dams and slopes were used to validate the trained structure. Subsequently, the classification algorithm was applied, and the outcome was compared against a set of annotated data. A few parameters were calculated and compared with the widely used algorithm for vegetation removal (i.e., CANUPO [22]) to provide an efficacy measurement. Those parameters were the confusion matrix, the number of 3D points of each misclassified bush, and computational load parameters (i.e., classification and training times, and computational load).

**Figure 5:**
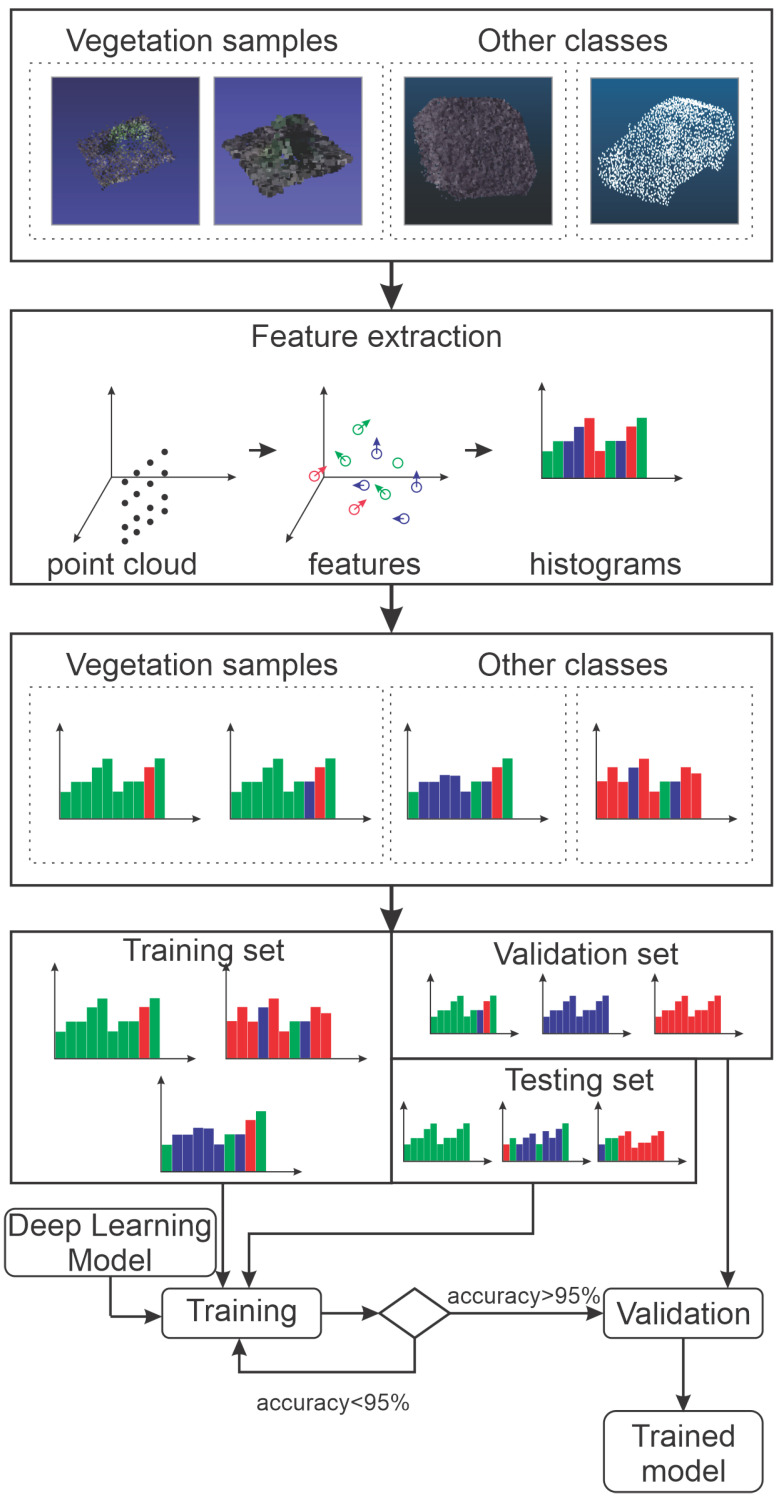
Experimental Process.

The experiment included two procedures. The first is the detection process that is shown in Figure 1, and the second the training, as shown in Figure 5. In the training process, vegetation samples and other classes are fed the feature extraction methods to produce proper input to the classification algorithm. Subsequently, the extracted features are randomly divided into training, validation, and testing sets at each trial. The training and test sets are used during the first stage in order to determine the algorithm accuracy. In a second stage, a validation set, never seen during the first stage, is classified to ensure that no bias was present during the first stages. The final products of the training process are the trained CNN models. The detection process was used in order to detect vegetation in the test images, and it is outlined in Figure 1.

## 3. Results and Discussion

### 3.1. Photogrammetry Survey

**Figure 6:**
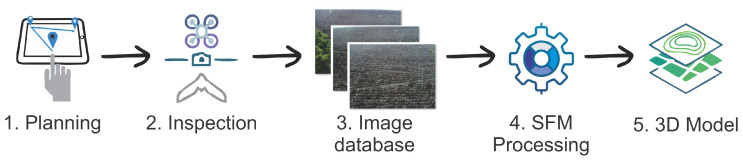
Aerial photogrametry inspection process.

In this work, an aerial photogrammetry survey was the data source for the 3D point clouds. The inspection process used can be summarized in five steps, as described in Figure 6. The mission was planned at each location following the inspected site using Google Earth data as a basis for mission planning [36,37]. This plan includes waypoint generation and ground control points for increased 3D point cloud accuracy. These points should remain in place for subsequent inspections increasing reconstruction accuracy.

The mission is executed at the site in the second step, and an image database is built. This image database consists of the images and their respective in-flight positions. Subsequently, SFM processing [8] is applied to rebuild the 3D point cloud from the respective surface. This step’s output is processed while using the methods described in this manuscript to obtain the 3D model from the surface.

The inspection process used in this work is only one of the methods that can be applied. Other methods, such as laser scan, could be deployed. In general, laser scan methods are exact and accurate to take distance measurements producing point clouds directly. Despite these clear advantages, they are not so common, due to the cost concerning image processing methods.

### 3.2. Test and Training Data

**Figure 7:**
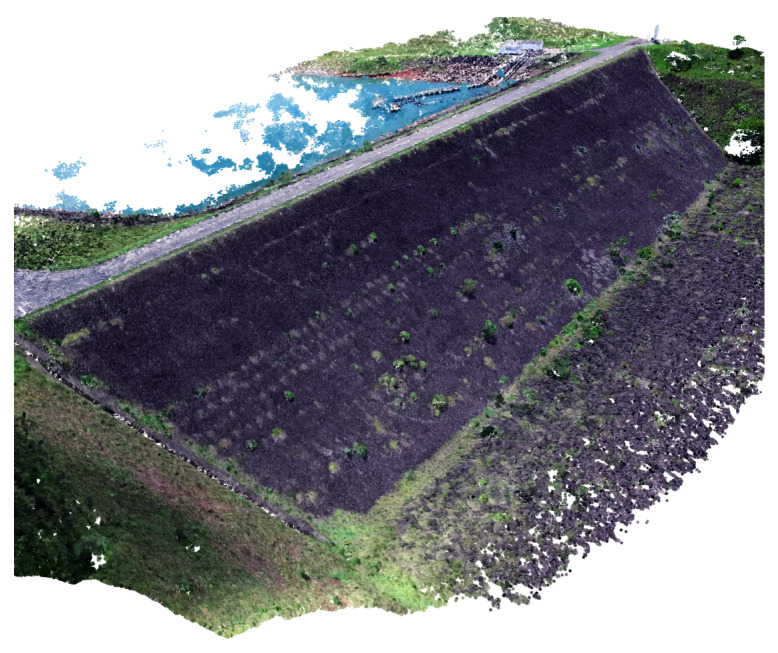
Three-dimensional Structure From Motion (3D SFM) Reconstruction of a Water Dam Inspection.

The data used in the experiments were gathered in a few locations, which are a water dam and slopes from other areas. Using the inspection procedure from Section 3.1, the images were gathered and 3D point clouds were built. Those reconstructions were used to analyze the vegetation removal accuracy. Note that any other method capable of generating 3D point clouds could have been used for 3D reconstruction Once the deep learning input from the proposed method is only the 3D cloud. Figure 7 illustrates the 3D reconstruction of a water dam inspection experiment.

**Figure 8:**
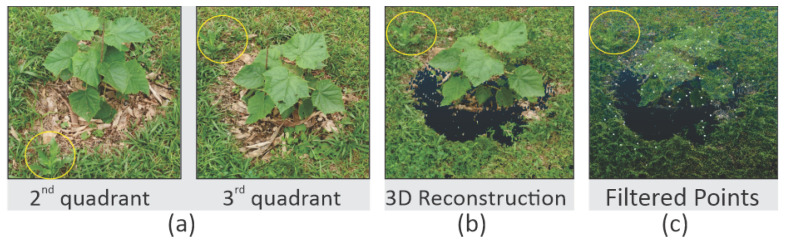
Example of Bush Reconstruction. (**a**) Images. (**b**) SFM. (**c**) After pre-processing.

A few other areas were inspected for building the training library of bushes. Note that only bushes were captures in 360 degrees to allow for a reasonable reconstruction of its characteristics. Figure 8a presents four quadrants of the same bush as an example of images and the resultant 3D image reconstruction. Figure 8b presents the dense cloud output. As described previously, the methodology applies a series of pre-processing steps. In Figure 8c, the pre-processing output is represented by the white points in the image. In the end, a text file containing the points positions and luminosity channel is exported for each bush. A second class containing a few examples of roads from real-world data was also built, aiming to provide a comparison for the Bush library.

**Figure 9:**
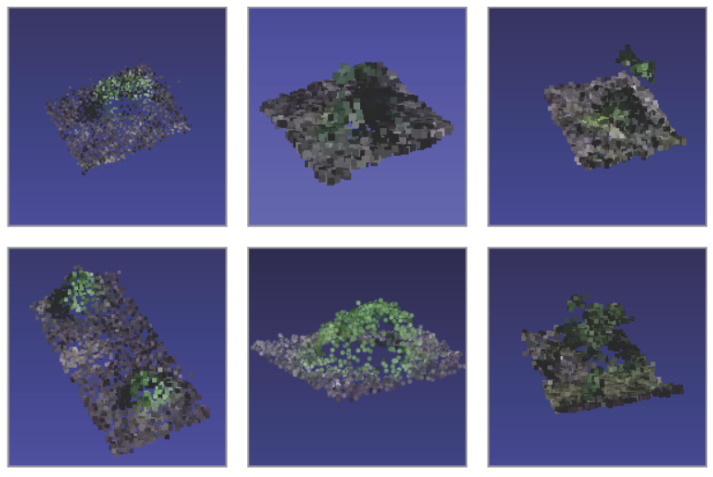
Sample of Bushes in The Library.

A library of bushes was built as a result of all processing stages, which is a representation of different types and plant species. However, it contains the main types that are found in the inspected places. Figure 9 presents a sample of the library. The original library with color and the full dense cloud is available in [38].

The library is used for training and testing the machine learning algorithm. A random selection of 15% of the library is separated at the start of each training section. In the end, real inspection data are used for algorithm validation. These two steps process ensure that over-fitting from the training library is not the cause for the algorithm performance.

The training process of the neural network is quite demanding. In our experiment, an intel i7 with 16 GB of memory and a Nvidea GTX1060 was used, and the training process demanded 12 h. However, using the trained model for the Water Dam and gravel inspection sites is less intensive and, to process each cloud, the same computer used only five minutes.

### 3.3. Neural Network

Table 1 shows the performance results for the proposed method as compared with other known methods of literature. These results are split for the test data with two and four classes. It is worth mentioning that the two-class example is a trained network that is designed to only recognize bushes and non-bushes classes. The four-class example is another trained network aimed at identifying the bush, car, people, and roads. Two and four classes were both run over test cases from the training database. These experiments intend to determine if a more general network is a better approach than a single class network. The other two results are related to the investigations in the real scenarios described before.

This table also presents the results for the real SFM reconstructions that were never seen by the proposed algorithm. For comparison, the SVM of CANUPO [22] was trained using the point cloud in the training library and then applied to these real reconstructions. It is possible to obtain better results when applying CANUPO if the algorithm is trained in the SFM reconstruction data. Note that this also happens with a neural network. The results are also obtained for a trained version of the PointNet model [23]. These results are also very similar to the outcomes that were obtained in our model. However, the proposed model has lower network complexity, which reduces the computational cost in the model.

**Figure 10:**
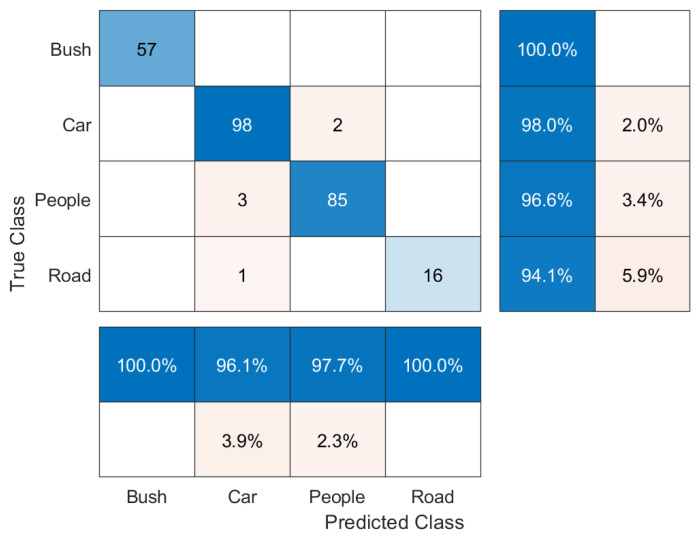
Classification Confusion Matrix.

The confusion matrix for the four-class experiment of Figure 1 is presented in Figure 10. This result allows for us to understand the algorithm performance regarding the input data in more detail. Note that the classes that use real data have a better performance. Additionally, real-world libraries would be necessary for determining what characteristics from the point cloud produce this advantage.

From the confusion matrix of Figure 10, it is possible to estimate the performance parameters. Table 2 presents the used formulas. Table 3 shows the results for each class. The basic terminology used in the confusion matrix is given by:

- Condition Positive (P): the number of real positive cases in the data;
- Condition Negative (N): the number of real negative cases in the data;
- True Positive (TP): condition positive detected as positive;
- True Negative (TN): condition negative detected as negative;
- False Positive (FP): equivalent with false alarm; and,
- False negative (FN): equivalent with miss.

Many different factors can affect the reconstruction quality and, consequently, these results. However, deep learning techniques tend to be quite robust to changes, such as orientation and illumination. However, the reconstruction quality should contain information at least to the point where the bush structure is clear. This means that dense point cloud with less than 0.02 m of a uniform sample.

**Figure 11:**
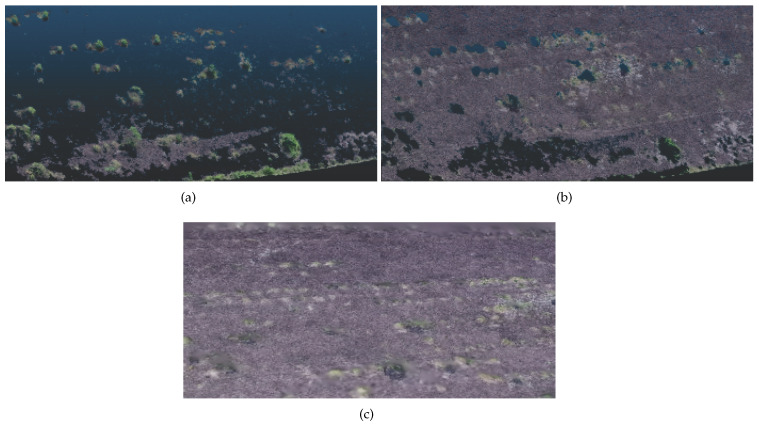
Results of the Algorithm on a Water Dam. (**a**) Vegetation. (**b**) Underlying soil. (**c**) Poisson surface reconstruction.

Figure 11 shows parts of the classification process applied in the water dam in order to demonstrate a qualitative representation of results. Note that parts of the soil were selected as vegetation. This is possible due to the result of the classification examples containing a piece of the soil around each bush. Despite the result, this is preferable for later analysis than having parts of the vegetation miss classified. Note that the latter, the surface, is rebuilt while using a Poisson surface reconstruction from oriented points, as shown in Figure 11c.

**Figure 12:**
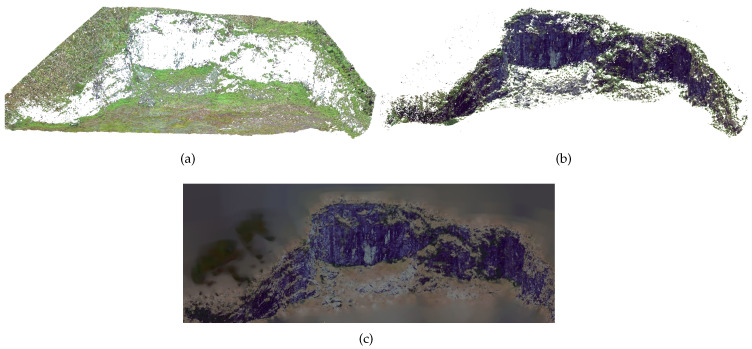
Results of the Algorithm Applied on an Abandoned Gravel Extraction Site. (**a**) Vegetation. (**b**) Underlying soil. (**c**) Poisson Surface Reconstruction.

**Figure 13:**
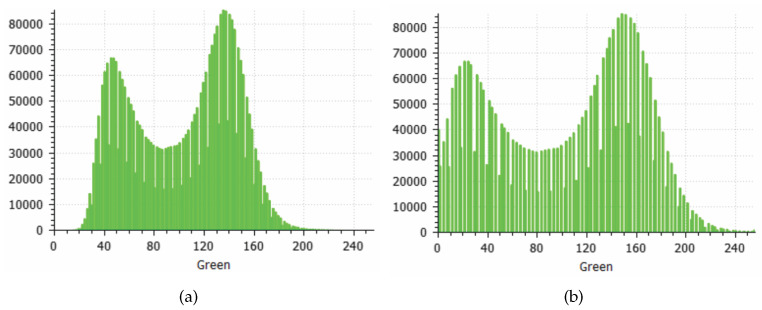
Green Channel Color Normalization. (**a**) Before. (**b**) After.

Figure 12 presents a similar case of the process. However, the location is an abandoned gravel extraction site, which may require regular safety monitoring inspections. This location was selected due to the differences in soil and vegetation presented by the water dam. Besides, it has a different type of vegetation with other characteristics, which makes the color more critical in the filtration process. An additional filter step is applied, as the grayscale representation drastically reduces the separability of the green and gray colors. Thus, the color channels from the original point cloud were normalized. Figure 13 illustrates this process. Note that, filtering the mean green color, the separability margin increases.

It is important to mention that a final filtration step was applied in the results of Figure 12 in order to remove the remaining vegetation, which also has not much texture due to the relatively low point-cloud density.

**Figure 14:**
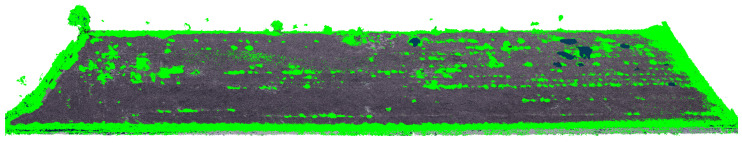
Classified vegetation marked in green.

A secondary result that was obtained by the method corresponds to the classified vegetation present on the scene. Figure 14 shows this result. Note that all of the points classified as vegetation are marked in green and they may not correspond to the original bush samples. This information can be used for processing in other methods, such as estimating the vegetation mass to be removed.

These results showed the method’s potential to classify parts of the scene. Trained models could be obtained for each object to be classified and used sequentially. Deep learning methods are more robust to change and they have a better performance than other machine learning methods, despite its computational cost.

**Figure 15:**
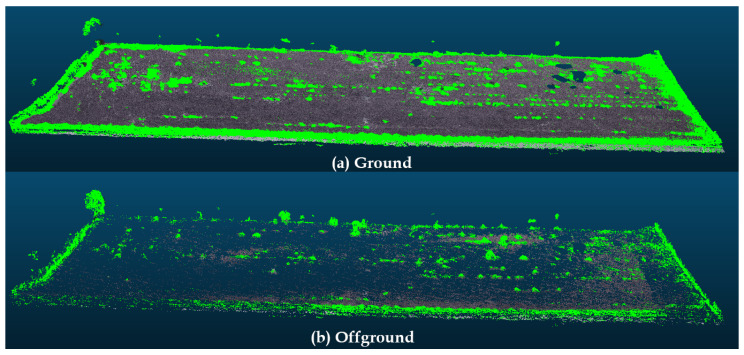
Classified vegetation show marked in green.

**Figure 16:**
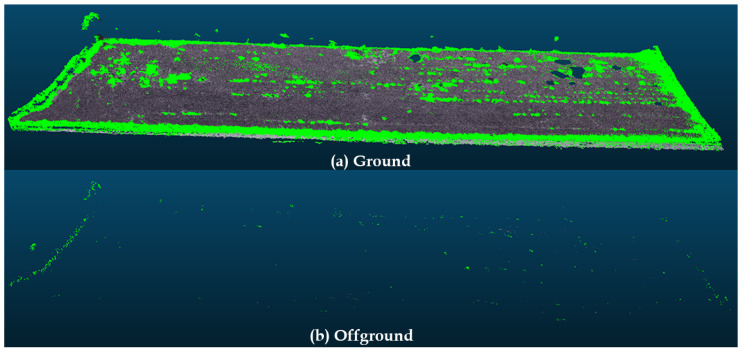
Classified vegetation show marked in green.

Here, a comparison with other methods to perform such task is also important. The cloth simulation filter is one of such methods [39,40]. This method is a 3D computer graphics algorithm, which is used for simulating cloth within a computer program. In order to compare the results, the input cloud from Figure 14 was processed while using CSF plugin from CloudCompare. Figure 15 shows the CSF result for 0.1 cloud resolution with 500 iterations and 0.2 of threshold. While Figure 16 used 0.1 cloud resolution with 1000 iterations and 0.5 of threshold. Note that many of the points were removed. However, the proposed method still has slightly better results than classifying the points to further processing later.

### 3.4. Deformation Analysis

The vegetation present in the point cloud is an issue for inspection methods. This is because the vegetation can change in shape or even move due to the wind and other causes. This movement will later be present in subsequent analysis, such as deformation and stress analysis.

**Figure 17:**
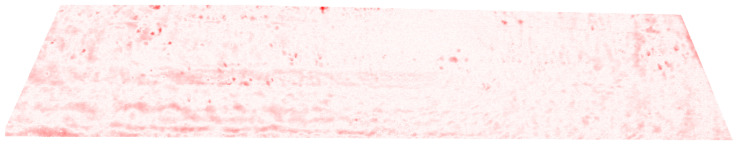
Second inspection at Water dam site.

The authors propose the displacement analysis for the point cloud in order to exemplify this scenario. To this end, the results that are shown in Figure 11 and reconstruction from a second inspection at the same site performed a few months later were used. The authors performed the displacement analysis. This consists of calculating the distance from the local surface from the first point cloud to the second. This process makes usage of the method described in [41]. Figure 17 shows this result where red areas indicate positive displacement, blue areas indicate negative displacement, and white areas indicate no displacement. More results that are related to this type of analysis can be found in [41].

## 4. Conclusions and Future Work

The presence of areas that are covered by vegetation makes the acquisition of an adequate object surface difficult, prejudicing the alignment estimation and soil movement calculation in 3D reconstructions. This work aims to reduce this effect by removing the vegetation that is present in the 3D reconstruction. The proposed methodology applies a combination of deep learning with a windowing processing in order to accurately determine the presence and position of vegetation that needs to be removed.

The process works, as follows. The output point cloud is smooth in a first filter stage due to large amounts of data at the center, and more spaced points are the edges. Subsequently, the classification occurs by applying a box due to the point cloud 3D position data. Afterwards, another filter stage removes the vegetation points that were leftover from the classification process. Note that the solution adopted in this research has presented good results when compared to other approaches in the current literature. The filtration steps are also very detailed and they can improve the outcomes for any similar work. Besides, this methodology represents a practical alternative approach for vegetation removal of complex natural scenarios.

It is worth noting that the representative sample used is still small and more experiments are required in order to improve the library size and increase the statistical significance of the result. The authors also acknowledge that mixing 3D data from real-world experiments with data from other libraries, such as MobileNet, can affect the method performance. However, libraries with similar data are still not widely available. A few improvements are expected for future works. For instance, it is intended to improve the library of examples to include other types of vegetation. Besides, different methodologies will be evaluated in this kind of application, such as the application of multi-spectral filtering that is based on thermal images and keypoint-based deep learning.

## Figures and Tables

**Table 1:** Classification Results.

Classification Method	Accuracy			
	2 Classes	4 Classes	Water Dam	Slopes
3DColored	0.98	0.97	0.93	0.90
3DUncolored	0.95	0.92	0.92	0.90
CANUPO SVM	-	-	0.89	0.78
PointNet	-	-	0.90	0.88

**Table 2:** Confusion Matrix Parameters.

**ACC**	$\frac{TP+TN}{TP+TN+FP+FN}$
**TPR**	$\frac{TP}{TP+FP}$
**TNR**	$\frac{TN}{TN+FN}$

**Table 3:** Confusion matrix parameters.

	TPR	TNR	ACC
Bush	93.4%	99.0%	100.0%
Car	96.1%	98.8%	96.1%
Road	80.0%	99.2%	100.0%
People	95.5%	98.8%	97.7%

## References

[1] Stumpf A., Malet J.P., Allemand P., Pierrot-Deseilligny M., Skupinski G. (2015). Ground-based multi-view photogrammetry for the monitoring of landslide deformation and erosion. Geomorphology.

[2] Barazzetti L., Scaioni M., Remondino F. (2010). Orientation and 3D modelling from markerless terrestrial images: Combining accuracy with automation. Photogramm. Rec..

[3] Yang H., Xu X., Neumann I. (2018). Optimal finite element model with response surface methodology for concrete structures based on Terrestrial Laser Scanning technology. Compos. Struct..

[4] Guisado-Pintado E., Jackson D.W., Rogers D. (2019). 3D mapping efficacy of a drone and terrestrial laser scanner over a temperate beach-dune zone. Geomorphology.

[5] Khaloo A., Lattanzi D., Jachimowicz A., Devaney C. (2018). Utilizing UAV and 3D Computer Vision for Visual Inspection of a Large Gravity Dam. Front. Built Environ..

[6] Pinto M.F., Marcato A.L., Melo A.G., Honório L.M., Urdiales C. (2019). A Framework for Analyzing Fog-Cloud Computing Cooperation Applied to Information Processing of UAVs. Wirel. Commun. Mob. Comput..

[7] Buffi G., Manciola P., Grassi S., Barberini M., Gambi A. (2017). Survey of the Ridracoli Dam: UAV–based photogrammetry and traditional topographic techniques in the inspection of vertical structures. Geomat. Nat. Hazards Risk.

[8] Nesbit P.R., Hugenholtz C.H. (2019). Enhancing UAV–SFM 3D model accuracy in high-relief landscapes by incorporating oblique images. Remote Sens..

[9] Walbridge S., Slocum N., Pobuda M., Wright D. (2018). Unified geomorphological analysis workflows with Benthic Terrain Modeler. Geosciences.

[10] Abellán A., Vilaplana J., Calvet J., García-Sellés D., Asensio E. (2011). Rockfall monitoring by Terrestrial Laser Scanning–case study of the basaltic rock face at Castellfollit de la Roca (Catalonia, Spain). Nat. Hazards Earth Syst. Sci..

[11] Vandapel N., Huber D.F., Kapuria A., Hebert M. Natural terrain classification using 3-d ladar data. Proceedings of the IEEE International Conference on Robotics and Automation (CRA’04).

[12] Plaza V., Ababsa F.E., Garcia-Cerezo A.J., Gomez-Ruiz J.A. 3d segmentation method for natural environments based on a geometric-featured voxel map. Proceedings of the 2015 IEEE International Conference on Industrial Technology (ICIT).

[13] Kragh M., Jørgensen R.N., Pedersen H. (2015). Object detection and terrain classification in agricultural fields using 3D lidar data. International Conference on Computer Vision Systems.

[14] Wen C., Sun X., Li J., Wang C., Guo Y., Habib A. (2019). A deep learning framework for road marking extraction, classification and completion from mobile laser scanning point clouds. ISPRS J. Photogramm. Remote Sens..

[15] Kumar A., Anders K., Winiwarter L., Höfle B. Feature Relevance Analysis for 3D Point Cloud Classification Using Deep Learning. Proceedings of the ISPRS Annals of the Photogrammetry, Remote Sensing and Spatial Information Sciences.

[16] Filippi A.M., Jensen J.R. (2007). Effect of continuum removal on hyperspectral coastal vegetation classification using a fuzzy learning vector quantizer. IEEE Trans. Geosci. Remote Sens..

[17] Gianinetto M., Lechi G. (2004). The development of superspectral approaches for the improvement of land cover classification. IEEE Trans. Geosci. Remote Sens..

[18] Natrajan P., Rajmohan S., Sundaram S., Natarajan S., Hebbar R. A Transfer Learning based CNN approach for Classification of Horticulture plantations using Hyperspectral Images. Proceedings of the 2018 IEEE 8th International Advance Computing Conference (IACC).

[19] Chen Y., Jiang H., Li C., Jia X., Ghamisi P. (2016). Deep feature extraction and classification of hyperspectral images based on convolutional neural networks. IEEE Trans. Geosci. Remote Sens..

[20] Zhao H., Zhang L., Zhao X. Mineral absorption feature extraction in vegetation covered region based on reference spectral background removal. Proceedings of the 2016 8th Workshop on Hyperspectral Image and Signal Processing: Evolution in Remote Sensing (WHISPERS).

[21] Sithole G., Vosselman G. (2004). Experimental comparison of filter algorithms for bare-Earth extraction from airborne laser scanning point clouds. ISPRS J. Photogramm. Remote Sens..

[22] Brodu N., Lague D. (2012). 3D terrestrial lidar data classification of complex natural scenes using a multi-scale dimensionality criterion: Applications in geomorphology. ISPRS J. Photogramm. Remote Sens..

[23] Qi C.R., Su H., Mo K., Guibas L.J. Pointnet: Deep learning on point sets for 3d classification and segmentation. Proceedings of the IEEE Conference on Computer Vision and Pattern Recognition.

[24] Panella F., Roecklinger N., Vojnovic L., Loo Y., Boehm J. (2020). Cost-Benefit Analysis of Rail Tunnel Inspection for Photogrammetry and Laser Scanning. Int. Arch. Photogramm. Remote Sens. Spat. Inf. Sci..

[25] Noordermeer L., Bollandsås O.M., Ørka H.O., Næsset E., Gobakken T. (2019). Comparing the accuracies of forest attributes predicted from airborne laser scanning and digital aerial photogrammetry in operational forest inventories. Remote Sens. Environ..

[26] Moon D., Chung S., Kwon S., Seo J., Shin J. (2019). Comparison and utilization of point cloud generated from photogrammetry and laser scanning: 3D world model for smart heavy equipment planning. Autom. Constr..

[27] Sharma N., Jain V., Mishra A. (2018). An analysis of convolutional neural networks for image classification. Procedia Comput. Sci..

[28] Yang J., Jiang Y.G., Hauptmann A.G., Ngo C.W. Evaluating bag-of-visual-words representations in scene classification. Proceedings of the International Workshop on Workshop on Multimedia Information Retrieval.

[29] Lampert C.H., Blaschko M.B., Hofmann T. Beyond sliding windows: Object localization by efficient subwindow search. Proceedings of the 2008 IEEE Conference on Computer Vision and Pattern Recognition.

[30] Seifert E., Seifert S., Vogt H., Drew D., Van Aardt J., Kunneke A., Seifert T. (2019). Influence of drone altitude, image overlap, and optical sensor resolution on multi-view reconstruction of forest images. Remote Sens..

[31] Union I.T. (2018). Report ITU-R BT.2380-2. https://www.itu.int/dms_pub/itu-r/opb/rep/R-REP-BT.2380-2-2018-PDF-E.pdf.

[32] Wu L.S., Wang G.L., Hu Y. (2020). Iterative closest point registration for fast point feature histogram features of a volume density optimization algorithm. Meas. Control.

[33] Li D., Liu N., Guo Y., Wang X., Xu J. (2019). 3D object recognition and pose estimation for random bin-picking using Partition Viewpoint Feature Histograms. Pattern Recognit. Lett..

[34] Wu Z., Song S., Khosla A., Yu F., Zhang L., Tang X., Xiao J. 3d shapenets: A deep representation for volumetric shapes. Proceedings of the IEEE Conference on Computer Vision and Pattern Recognition.

[35] Yi L., Kim V.G., Ceylan D., Shen I., Yan M., Su H., Lu C., Huang Q., Sheffer A., Guibas L. (2016). A scalable active framework for region annotation in 3d shape collections. ACM Trans. Graph. (TOG).

[36] Pinto M.F., Honorio L.M., Melo A., Marcato A.L. (2020). A Robotic Cognitive Architecture for Slope and Dam Inspections. Sensors.

[37] Pinto M.F., Honório L.M., Marcato A.L., Dantas M.A., Melo A.G., Capretz M., Urdiales C. (2020). ARCog: An Aerial Robotics Cognitive Architecture. Robotica.

[38] Melo A.G., Pinto M.F., Honório L.M. (2019). BushDataset, a Library of 3d Bushes to Machine Learning Applications. https://github.com/ARCog/BushDataset.

[39] Zhang W., Qi J., Wan P., Wang H., Xie D., Wang X., Yan G. (2016). An easy-to-use airborne LiDAR data filtering method based on cloth simulation. Remote Sens..

[40] Cai S., Zhang W., Liang X., Wan P., Qi J., Yu S., Yan G., Shao J. (2019). Filtering airborne LiDAR data through complementary cloth simulation and progressive TIN densification filters. Remote Sens..

[41] Melo A.G., Pinto M.F., Honório L.M., Dias F.M., Masson J.E.N. (2020). 3D Correspondence and Point Projection Method for Structures Deformation Analysis. IEEE Access.

